# A Rapid and Accurate Quantitative Analysis of Cellulose in the Rice Bran Layer Based on Near-Infrared Spectroscopy

**DOI:** 10.3390/foods12162997

**Published:** 2023-08-09

**Authors:** Shuang Fan, Chaoqi Qin, Zhuopin Xu, Qi Wang, Yang Yang, Xiaoyu Ni, Weimin Cheng, Pengfei Zhang, Yue Zhan, Liangzhi Tao, Yuejin Wu

**Affiliations:** 1 Anhui Key Laboratory of Environmental Toxicology and Pollution Control Technology, Hefei Institutes of Physical Science, Chinese Academy of Sciences, Hefei 230031, China; fans0723@mail.ustc.edu.cn (S.F.); cqqin@mail.ustc.edu.cn (C.Q.); xuzp@iim.ac.cn (Z.X.); wangqi@ipp.ac.cn (Q.W.); yangyang19860207@ipp.ac.cn (Y.Y.); nixiaoyu@iim.ac.cn (X.N.); cwm1210@mail.ustc.edu.cn (W.C.); pfzhang@aiofm.ac.cn (P.Z.); zhany@ipp.ac.cn (Y.Z.); mr.taoliangzhi@gmail.com (L.T.); 2 Science Island Branch, Graduate School of USTC, Hefei 230026, China; 3 Hainan Branch of the CAS Innovative Academy for Seed Design, Sanya 572019, China

**Keywords:** cellulose content, rice bran layer, near-infrared spectroscopy, diffuse reflectance, diffuse transmittance, fused spectra

## Abstract

Cultivating rice varieties with lower cellulose content in the bran layer has the potential to enhance both the nutritional value and texture of brown rice. This study aims to establish a rapid and accurate method to quantify cellulose content in the bran layer utilizing near-infrared spectroscopy (NIRS), thereby providing a technical foundation for the selection, screening, and breeding of rice germplasm cultivars characterized by a low cellulose content in the bran layer. To ensure the accuracy of the NIR spectroscopic analysis, the potassium dichromate oxidation (PDO) method was improved and then used as a reference method. Using 141 samples of rice bran layer (rice bran without germ), near-infrared diffuse reflectance (NIRdr) spectra, near-infrared diffuse transmittance (NIRdt) spectra, and fusion spectra of NIRdr and NIRdt were used to establish cellulose quantitative analysis models, followed by a comparative evaluation of these models’ predictive performance. Results indicate that the optimized PDO method demonstrates superior precision compared to the original PDO method. Upon examining the established models, their predictive capabilities were ranked in the following order: the fusion model outperforms the NIRdt model, which in turn surpasses the NIRdr model. Of all the fusion models developed, the model exhibiting the highest predictive accuracy utilized fusion spectra (NIRdr-NIRdt (1st der)) derived from preprocessed (first derivative) diffuse reflectance and transmittance spectra. This model achieved an external predictive R^2^_p_ of 0.903 and an RMSEP of 0.213%. Using this specific model, the rice mutant O2 was successfully identified, which displayed a cellulose content in the bran layer of 3.28%, representing a 0.86% decrease compared to the wild type (W7). The utilization of NIRS enables quantitative analysis of the cellulose content within the rice bran layer, thereby providing essential technical support for the selection of rice varieties characterized by lower cellulose content in the bran layer.

## 1. Introduction

Rice serves as the staple food for over half of the global population, accounting for a significant proportion of human calorific intake [1]. With socioeconomic development and improving living standards, there has been a growing demand for deep-milled, polished white rice among consumers. However, this consumer trend has induced an over-milling issue within the rice processing industry [2], resulting in substantial food waste and posting a threat to global food security. According to Dhankhar’s study [3], commercial rice mills can lose up to 25% of rice weight during milling. Reducing such losses is thus crucial to ensuring global food security. Moreover, over-milling compromises rice’s nutritional value by discarding the bran layer and germ, which contain vital nutrients, such as protein, lipids, dietary fiber, and vitamins [4]. The removal of the bran layer and germ renders milled rice nutritionally inferior to brown rice, adversely affecting human health [5,6,7]. Although brown rice has better nutritional value and health benefits than milled rice, consumer acceptance of brown rice remains low due to its perceived inferior palatability and texture [8]. This is attributed to the bran layer’s dense fibrous structure, which inhibits the starch components’ water absorption and gelatinization during cooking [9,10]. Previous research has demonstrated that the degradation of cellulose in the rice bran layer via cellulose enzymes can soften the texture of cooked brown rice, thereby enhancing its palatability [11,12,13]. This could be attributed to cellulose being a key component of the dense fiber structure in the bran layer, and its degradation contributes to the destruction of this dense structure. However, recent research suggests that breeding rice varieties with reduced Insoluble Dietary Fiber/Soluble Dietary Fiber (IDF/SDF) in the bran layer could be beneficial for improving the texture of brown rice [14]. As cellulose is an important type of IDF in brown rice, breeding rice varieties with reduced cellulose content in the bran layer could likely improve the texture of brown rice. Furthermore, studies have found a negative correlation between cellulose content and digestibility [15], suggesting that bran layer cellulose content could be a significant indicator of brown rice’s digestibility and quality. Therefore, exploring rice genetic germplasm with reduced cellulose content in the bran layer may enhance brown rice’s palatability and digestibility, offering a new technological avenue for optimizing brown rice utilization and bolstering food security.

The fundamental requirement for breeding rice varieties with low cellulose content in the bran layer is the swift and precise quantification of cellulose content. However, the rice bran layer is a structure tightly wrapped around the outside of the rice seeds’ endosperm, rendering the separation of an intact rice bran layer extraordinarily challenging. Rice bran, a by-product generated during the procession from brown rice to milled rice, is primarily constituted of the bran layer, germ, and a small amount of endosperm. When endosperm content is negligible, rice bran samples from which the germ has been removed can be regarded as the bran layer for investigative purposes. Moreover, conventional wet chemical analysis methods employed for determining plant cellulose content, including the Van Soest method [16], anthrone colorimetry [17], high-performance liquid chromatography [18], and potassium dichromate oxidation [19], bear high costs and are time-intensive, making them unfit for large-scale rice bran layer cellulose germplasm screening. Therefore, the predominant technical constraint in the screening of rice bran layer cellulose germplasm is the pressing necessity for a non-destructive, rapid, and precise method of cellulose content analysis.

Near Infrared Spectroscopy (NIRS) has gained prominence in agriculture [20], food [21], petrochemicals [22], and medicine [23] for its ability to perform rapid, cost-effective, and non-destructive quantitative and qualitative analyses. Numerous studies have utilized NIRS for the quantitative analysis of plant cellulose. For instance, Wang et al. [24] successfully developed a quantitative model for corn straw cellulose using NIRS, obtaining a determination coefficient (R^2^) and a root mean square error of prediction (RMSEP) of 0.968 and 0.683%, respectively. Similarly, Nielsen et al. [25] used NIRS for the quantitative analysis of cellulose content in wheat straw., reporting R^2^ and RMSEP values of 0.88 and 1.14%, respectively. Belen et al. [26] conducted quantitative analyses of cellulose in various grain samples, such as corn, wheat, and barley, using NIRS. Their model reported an R^2^ of 0.70 and an RMSEP of 0.98%. These studies attest to the potential of NIRS in the quantitative analysis of straw and grain cellulose. However, the application of NIRS for quantitative analysis of cellulose in the rice bran layer remains largely unexplored.

In contrast to straw, which can contain up to 30% cellulose, rice grains have considerably less. As noted by Gloria et al. [15], the cellulose content in brown rice and rice bran was reported as 0.11% and 4.39%, respectively. This discrepancy poses a challenge to the use of NIRS for accurate cellulose quantification in the rice bran layer. To improve the accuracy of NIRS, it is crucial to choose a high-precision reference method and an appropriate measurement mode, such as diffuse reflection (DR) mode or diffuse transmission (DT) mode. Furthermore, the accuracy of NIR spectroscopic analysis can be enhanced by employing data fusion technology, which combines data from disparate sources. As the sourced information might be redundant or complementary, the fused data tends to be more reliable and accurate compared to single-source data. For instance, Xu et al. [27] validated the viability of utilizing the data fusion method to achieve high-accuracy NIR spectroscopic analysis for rice flour. Nevertheless, to date, no instances of data fusion technology application in determining rice bran layer components have been documented.

In this study, the potassium dichromate oxidation (PDO) method—a standard wet chemical analysis procedure for determining cellulose content—was initially optimized and utilized as the reference method for NIR spectroscopic analysis. Utilizing near-infrared diffuse reflectance (NIRdr) and diffuse transmission (NIRdt) spectra, we established quantitative models for measuring cellulose content in the rice bran layer, respectively. Moreover, this study demonstrated the viability of boosting model accuracy through the fusion of NIRdr and NIRdt spectral data. We conducted a comparative analysis of the predictive capabilities of the NIRdr, NIRdt, and their fused model under various optimization conditions concerning the cellulose content in the rice bran layer and assessed their potential in screening for rice germplasm with low cellulose concentrations in the bran layer. Notably, the rice bran layer samples used in this study are rice bran without germ. This study aims to develop an accurate, non-destructive, and cost-effective method for quantifying cellulose content in the rice bran layer using NIRS technology, thereby offering substantial technical support for the selection of rice genetic germplasm and the cultivation of varieties with lower cellulose content in the rice bran layer.

## 2. Materials and Methods

### 2.1. Materials

**Figure 1:**
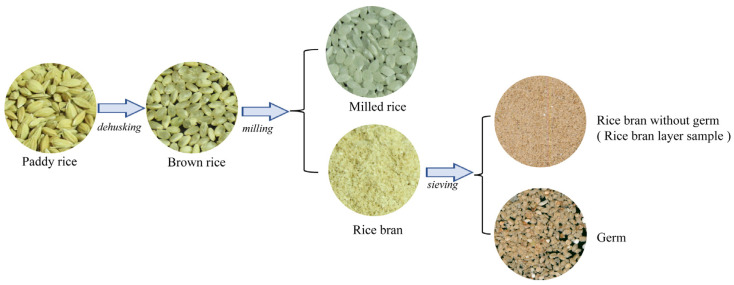
The general acquisition process of rice ran layer sample.

The rice bran layer samples used in this study were rice bran with germ removed by a 40-mesh sieve. A general overview of the acquisition process is presented in Figure 1. For a comprehensive description of the procedure used to obtain the rice bran layer samples, please refer to Part 1 of the Supplementary Material. A set of 141 rice bran layer samples derived from various rice germplasm and breeding lines was used to constitute the rice bran layer CC dataset. These rice germplasms and breeding lines were selected from the rice mutant repository constructed by our laboratory. The mutant repository was composed of the mutant offspring of rice varieties ‘9311’ and ‘Wuyunjing 7’, which were irradiated with low-energy heavy ions and cultivated for multiple generations [28,29]. By using the Kennard–Stone algorithm [30], the first 70% of the samples in the rice bran layer CC dataset were classified as the calibration set, and the remaining 30% was classified as the validation set. In addition, to further evaluate the prediction performance of the model established in this study, 20 rice bran layer samples not belonging to the calibration set and validation set were randomly selected from the above mutant repository as the external prediction set.

### 2.2. Chemical Analysis

#### 2.2.1. Determination Method of Cellulose

The content of cellulose was determined by an optimized chemical method. This optimized method is based on the PDO method, which was initially proposed by Halliwell [19] and then further developed by Xiong et al. [31]. The optimization of this method was performed by our laboratory. The general procedure of the optimized PDO method was the same as the original PDO method, including digestion, centrifugation, washing, oxidation reaction, and titration. However, some details have been improved. The specific improvements are as follows: (1) The centrifugal system was changed from 50 mL to 10 mL, which solved the problem of poor precipitation effect of rice bran layer sample digestion products in the 50 mL centrifugal system. (2) The primary and secondary centrifugal system was established. The supernatant, which should have been discarded in the original method, was put into a new 10 mL centrifuge tube (secondary centrifuge tube) for centrifugal treatment, and the precipitation was incorporated into the main centrifuge tube, which reduced the loss of precipitation and improved the detection accuracy. (3) The dosage of the potassium dichromate-sulfuric acid mixture was adjusted from 18 mL to 5 mL; (4) The equivalent concentration of ammonium ferrous sulfate in the titration solution was adjusted from 0.1 N to 0.05 N, which reduced the titration error and further improved the detection accuracy. The specific steps of the optimized PDO method are shown in Part 2 of the Supplementary Material.

#### 2.2.2. Validation of the Optimized PDO Method

The validation of the optimized PDO method was performed on the following two aspects: (1) The evaluation of intra-day precision and inter-day precision of the optimized PDO method for the determination of cellulose content in the rice bran layer; (2) the comparison between the determination results of the optimized PDO method and the original PDO method.

The intra-day and inter-day precision of the optimized PDO method was evaluated on the same rice bran layer sample, which was derived from the rice variety (K7). Among them, the intra-day precision of cellulose content measurements was calculated as the relative standard deviation (RSDr) of 6 consecutive measurements of the rice bran layer sample within the same day. Similarly, the inter-day precision of cellulose content measurements was calculated as the relative standard deviation (RSDR) of 18 measurements of the rice bran layer sample over three days of analysis [32,33,34]. Notably, both intra-day and inter-day precision characterize the repeatability of the method, as they were confirmed through experiments conducted by the same experimenter using identical equipment [35].

To compare the determination results of the optimized PDO method with those of the original PDO method, 7 rice bran layer samples were assessed using the optimized PDO method and the original PDO method. Three repeated experiments were set up for each method. For a certain sample, the average value of three repeated experiments was taken as the cellulose content determination result of the corresponding method. The standard deviation (SD) and relative standard deviation (RSD) of three repeated measurements of each sample were calculated to compare the precision of the optimized PDO method and the original PDO method. In addition, OriginPro Software v.8.5 (OriginLab Corp., Northampton, MA, USA) was used to analyze the correlation between the determination results of the optimized PDO method and the original PDO method.

### 2.3. NIR Spectroscopy Measurement

**Figure 2:**
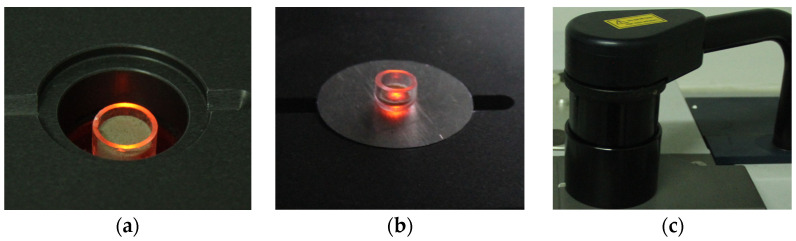
Schematic diagram of diffuse reflection (**a**) and diffuse transmission (**b**,**c**) spectra acquisition.

All of the spectra were recorded on an MPA Fourier transform near-infrared spectrometer (Bruker, Ettlingen, Germany), which supports two measurement modes: diffuse reflection and diffuse transmission. The NIRdr spectra, which were obtained under the diffuse reflection mode, were acquired in the range of 4000–12,000 cm^−1^ (833.3–2500 nm) with a resolution of 16 cm^−1^ and 32 repeat scans. The NIRdt spectra, which were obtained under the diffuse transmission mode, were acquired in the range of 5793–12,489 cm^−1^ (800.7–1726.2 nm) with a resolution of 16 cm^−1^ and 64 repeat scans. The schemata of the NIR spectra acquisition method for the dataset are shown in Figure 2.

The NIRdr spectral acquisition method for rice bran layer samples was as follows: Each rice bran layer sample was placed in a cylindrical sample cup, which was a quartz glass dish with an 11.5 mm diameter and a 7 mm height, to prevent interferences in its absorption. Once the sample cup containing the sample was placed in the sample window, the NIRdr spectrum would be recorded under the diffuse reflectance mode (Figure 2a). Every sample underwent a single-spectrum recording.

The NIRdt spectral acquisition method for rice bran layer samples was as follows: Each rice bran layer sample was weighed (10 mg) and placed in a cylindrical sample cup, which was a quartz glass dish with an 8 mm diameter and a 5 mm height. The sample was gently pressed with a metal rod to ensure it was evenly spread at the bottom of the sample cup. The thickness of the sample has been validated through the thickness test mentioned in the study by Xiang et al. [36] (for details, please refer to Part 3 of the Supplementary Material). The purpose of this procedure is to ensure the sample is thin enough so that near-infrared radiation can be transmitted through the sample to the sensor. An aluminum sheet, which has a central hole with a diameter of 2 mm, was positioned over the sample window. Once the sample cup containing the sample was positioned over the aluminum sheet, the NIRdt spectrum would be recorded under the diffuse transmission mode (Figure 2b). The aluminum sheet, acting as a smaller collection window, ensured that only near-infrared radiation passing through the hole was recorded. This design restricted the collected data solely to the sample, mitigating background interference resulting from variations in the size of the sample cup containing the sample, thus enhancing the consistency of transmittance spectra collection. Every sample underwent a single-spectrum recording.

It should be noted that the detector used in the diffuse reflectance mode is located inside the instrument and is used to receive the near-infrared radiation diffusely reflected from the sample, whereas the detector used in the diffuse transmittance mode is located on the outside of the instrument, used for receiving the near-infrared radiation transmitted through the sample. When using the diffuse transmittance mode to acquire spectra, it is necessary to manually rotate the outside detector to the top of the detection window and cover the sample, as shown in Figure 2c.

### 2.4. The Construction and Evaluation of the NIR Model

To obtain a calibration model for cellulose, chemometric analysis was performed using the OPUS 7.0 software (Bruker, Ettlingen, Germany). Before the development of calibration models, spectral data were pre-treated to reduce the interference of useless information and noise in the spectra. The spectral pre-treatment methods used in this study included first-order derivatives (1st der, with 17-point smoothing by default), multiplicative scattering correction (MSC), standard normal variate transformation (SNV), the 1st der combined with the MSC method (1st der + MSC) and the 1st der combined with the SNV method (1st der + SNV). The MSC and SNV methods both aim to correct the spectral scattering effect instigated by factors including particle size and shape [37]. These two methods can frequently be combined with other pretreatment methods, such as 1st der, thereby enhancing the effectiveness of spectral pretreatment.

In addition to selecting an appropriate pretreatment method, it is also necessary to select the appropriate spectral range through specific methods to construct a high-quality calibration model. On the one hand, the selection of spectral range can improve the operation efficiency and interpretability of the model. On the other hand, because the irrelevant variables are eliminated in the process of selecting the spectral range, the calibration model with strong prediction ability and good robustness can be obtained [38]. In general, the spectral data pre-treated methods need to be compared or combined, and the partial least squares (PLS) model is constructed under the combined conditions of each pretreatment method and spectral range.

In this study, the optimization function of the OPUS 7.0 software (Bruker, Ettlingen, Germany) was used to screen the optimal combined conditions of the pre-treated method and spectral range to optimize the calibration model. This function is based on the principle of the siPLS algorithm. The core procedure of this function is to divide the pre-treated spectrum into 10 segments at equal intervals and randomly combine 1–6 segments to construct a calibration model [39].

In the process of model construction, it is necessary to use the calibration set to perform the cross-validation of the model, and the quality of the model is evaluated by the root mean square error of cross-validation (RMSECV) and the coefficient of determination of cross-validation (R^2^_cv_) to select the optimal modeling parameters. Generally, the model with the lowest RMSECV value and the highest R^2^_cv_ value was used as the optimal model. After the model was established, it was necessary to use the validation set to verify the accuracy and robustness of the model. The model with a low root mean square error of validation (RMSEV) value and a high coefficient of determination of validation (R^2^_v_) value usually had high accuracy. In addition, this study also used an external prediction set which is independent of the calibration set and the validation set to test the prediction performance of the model. The model with the lowest root mean square error of prediction (RMSEP) value and the highest coefficient of determination of prediction (R^2^_p_) had the best prediction performance. Statistical analysis and graphing were conducted with OriginPro Software (OriginLab Corp., Northampton, MA, USA).

### 2.5. Fusion of NIRdr and NIRdt Spectra

The fusion method refers to the method reported by Xu et al. [27]. Before data fusion, in order to correct the difference in absorbance between NIRdr and NIRdt spectra, three different pretreatment methods (no pretreatment, min-max normalization, and 1st der) were used to process the NIRdr and NIRdt spectra, respectively. The NIRdr and NIRdt spectra of each sample which were processed by the same pretreatment method (no pretreatment, min-max normalization, or 1st der), were then directly joined using the primary fusion method to obtain the fused spectra. According to the pretreatment method (no pretreatment, min-max normalization, or 1st der) used by the components (NIRdr and NIRdt spectra) of the fused spectrum, the fusion spectrum was recorded as NIRdr-NIRdt, NIRdr-NIRdt (NM) or NIRdr-NIRdt (1st der). MATLAB software (MathWorks, Natick, MA, USA) was used to obtain the fused spectra.

### 2.6. Statistical Analysis Software

Statistical analyses performed in this study, such as correlation analysis, paired *t*-tests, and independent-sample *t*-tests, were all conducted using the OriginPro Software (OriginLab Corp., Northampton, MA, USA).

## 3. Results and Discussion

### 3.1. Validation of the Optimized PDO Method

#### 3.1.1. Intra-Day Precision and Inter-Day Precision of the Optimized PDO Method

Table 1 presents the intra-day precision RSDr calculated within each day of analysis on six measurements performed on the same rice bran layer sample using the optimized PDO method. The optimized PDO method demonstrated good repeatability within the same day, as indicated by the low RSDr, ranging from 0.51% (Day 2) to 0.96% (Day 1); all values were less than 2%. Inter-day precision RSDR, calculated across three days of analyses on 18 measurements performed on the same rice bran sample using the optimized method, was 0.70% (Table 1). The results showed that the optimized PDO method had a good inter-day precision performance.

#### 3.1.2. Comparison between the Optimized PDO Method and the Original PDO Method

The optimized PDO method and the original PDO method were used to repeatedly determine fifteen rice bran layer samples three times, respectively. Table 2 recorded the mean value, standard deviation (SD), and relative standard deviation (RSD) of the determination results of each sample. The detailed results of the determination are presented in Part 4 of the Supplementary Material.

**Figure 3:**
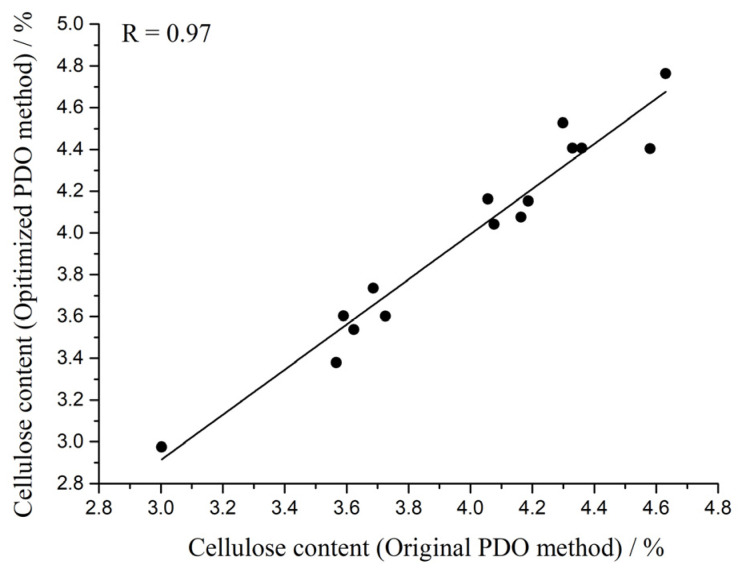
Correlation detection between the optimized PDO method and the original PDO method.

Based on the mean value recorded in Table 2, the correlation between the optimized PDO method and the original PDO method was analyzed by statistical methods. As shown in Figure 3, the correlation coefficient (R) between the two methods is 0.97, which indicates that there is a good correlation between the optimized PDO method and the original PDO method. A two-tailed *t*-test was performed between the mean values obtained by the optimized PDO method and the mean values obtained by the original PDO method to evaluate the significant relationship between the optimized PDO method and the original PDO method. The t-value was 0.22, less than the value of t_14,0.05_ (t_14,0.05_ = 2.14, two-tailed test), and the *p*-value was 0.83, greater than 0.05, indicating that there was no significant difference between the optimized PDO method and the original PDO method. Combined with the results of correlation analysis, it can be seen that the optimized PDO method has the potential to replace the original PDO method for cellulose determination.

RSD is often used to characterize the precision of the method, and its calculation formula is RSD = SD/Mean*100%. According to Table 2, when measuring the cellulose content of the above 15 rice bran layer samples, the mean RSD of the optimized PDO method is 1.36%, while the mean RSD of the original PDO method is 2.80%. It can be inferred that the precision of the optimized PDO method is higher than that of the original PDO method. To verify the above inference, the RSD values of the optimized PDO method and the original PDO method were compared by a one-tailed *t*-test. After testing, the t-value was 4.57, which was greater than the t_14,0.01_ value (t_14,0.01_ = 2.62, single tail test), and the *p*-value was 0.00022, which was less than 0.01, indicating that the precision of the optimized PDO method was indeed higher than that of the original PDO method. In summary, the optimized PDO method is more suitable as a reference method than the original PDO method, which will help improve the prediction performance of near-infrared spectroscopy.

### 3.2. Results of Chemical Determination of Rice Bran Layer Sample Set

The statistical results of the cellulose content in rice bran layer samples determined using the optimized method are shown in Table 3. The distribution of cellulose content in the calibration set ranged from 2.80% to 4.92%, which completely covered the range of the validation set. The mean values and standard deviations (SD) of the calibration set and validation set were similar, indicating that these two data sets show similar distribution characteristics and measures of central tendency. The standard error (SE) in Table 3 is derived by dividing the SD by the square root of the sample number (N). The confidence intervals (mean ± SE) for the mean cellulose reference values in the calibration and validation sets are 3.836–3.944% and 3.744–3.916%, respectively. The overlapping intervals suggest no significant difference between these two datasets. This is confirmed by an independent-samples *t*-test on the cellulose content reference values of these two datasets, resulting in a *p*-value of 0.55, which indicates no significant difference between the calibration set and the validation set. These results show that the calibration set is a good representative of the validation.

### 3.3. Fused Spectra Obtained under Three Different Pretreatment Conditions

**Figure 4:**
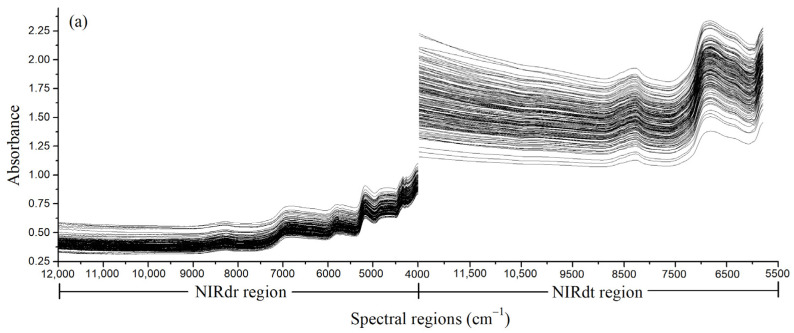

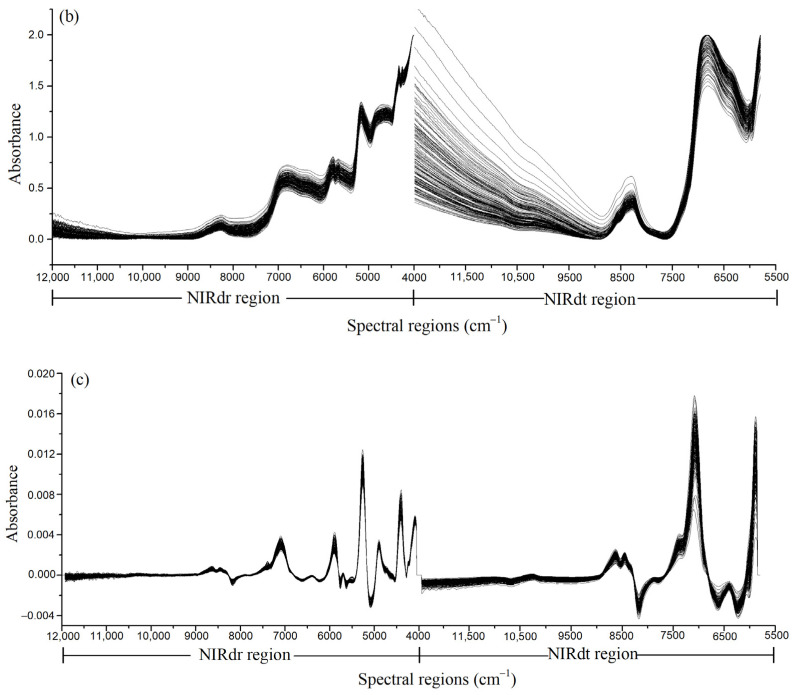
(**a**) NIRdr-NIRdt, (**b**) NIRdr-NIRdt (NM), and (**c**) NIRdr-NIRdt (1st der) spectral profiles recorded for the rice bran layer samples.

The fused NIRdr and NIRdt spectra derived from the rice bran layer sample set under the three pretreatment conditions are shown in Figure 4. The left part of each fusion spectrum is the NIRdr spectrum, and the right part is the NIRdt spectrum. All spectra in the figure are displayed in wavenumber format.

As depicted in Figure 4a, the NIRdr-NIRdt spectral profile’s two sections, namely the NIRdr and NIRdt regions, displayed distinct absorbance values. Both the NIRdr and NIRdt regions showed absorption peaks at 8330 cm^−1^ (1200 nm) and 6825 cm^−1^ (1465 nm), which correspond to the characteristic absorption bands of the second overtone of the C–H stretch vibration and the first overtone of O–H stretch vibration, respectively. Furthermore, both of them are related to the spectral absorption of cellulose [40]. Within the 3996–5793 cm^−1^ (1726–2502 nm) interval in the NIRdr region, there is the first overtone of the C–H stretch vibration and the first combination bands of C–H and O–H stretch vibrations, which are related to the spectral absorption of cellulose [41]. However, this range is absent in the NIRdt region. The differences observed in spectral profiles and ranges imply that the NIRdt spectrum tends to collect the sample’s signal characteristics in the short-wavelength regions, while the NIRdr spectra exhibit higher sensitivity to absorption within the long-wavelength regions. Moreover, the absorbances measured in the NIRdt spectra exhibited a higher magnitude compared to those recorded in the NIRdr spectra. The main reason is that the near-infrared radiation transmittance of the rice bran layer sample is limited, resulting in less near-infrared radiation returning to the sensor in the transmission mode. Due to the difference in absorbance, there is an obvious gap at the junction of the NIRdr and NIRdt spectra.

In Figure 4a, significant baseline drift is observed in the NIRdt region, which may be attributed to the variations in sample density within the sample cup. Different sample densities imply differences in the distances between sample particles, resulting in notable discrepancies in the transmitted signal through the sample. In contrast, the NIRdr region exhibits a smaller baseline drift, likely due to the limited penetration of the reflected signal that comes back to the sensor in the reflection mode, resulting in less sensitivity to sample density. The baseline drift phenomenon can be corrected through appropriate spectral pretreatment methods. 

For the NIRdr-NIRdt (NM) spectrum (Figure 4b), the normalization pretreatment method was used to correct the differences in the absorbance between the NIRdr and NIRdt regions to a similar scale. At the same time, the baseline drift phenomenon had also been improved. However, the baseline drift of the NIRdt region in the range of 9500–12,500 cm^−1^ had not been effectively improved.

For the NIRdr-NIRdt (1st der) spectrum (Figure 4c), the derivative pretreatment method was employed to alter the trend of absorbance recorded in the NIRdr and NIRdt regions with respect to wavenumber. Under this pretreatment condition, the absorbance of the fused spectra exhibited oscillations around the zero baseline. In the NIRdr-NIRdt (1st der) spectral profiles, obvious peaks and troughs were observed in the regions exhibiting strong characteristic absorption, whereas the absorbance values in other regions approached zero. It can be seen from Figure 4c that the gap at the splicing between NIRdr and NIRdt spectra can be effectively reduced by using the derivative pretreatment method.

### 3.4. NIR Calibration and Validation Results

**Figure 5:**
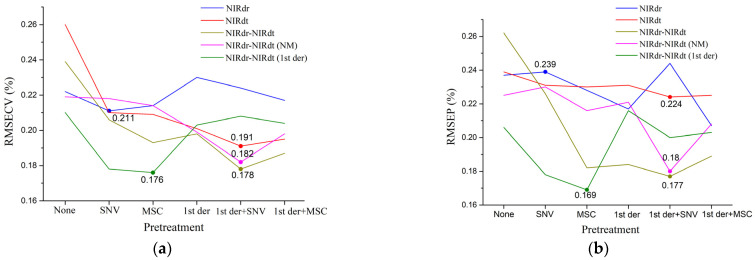
Comparison of (**a**) RMSECV and (**b**) RMSEP for models under six pretreatment Conditions. Different colored lines represent models constructed based on distinct types of spectra. Dots on each line, as illustrated in (**a**,**b**), represent the RMSECV and RMSEP of the optimal model constructed based on the corresponding type of spectra with specific numerical values.

PLS models were established based on NIRdr, NIRdt, NIRdr-NIRdt, NIRdr-NIRdt (NM), and NIRdr-NIRdt (1st der) spectra, respectively. Figure 5 offers a visual comparison of the RMSECV and RMSEP values for these models under six different pretreatment conditions. Each type of spectra has an associated optimal model, the RMSECV value of which is prominently represented by dots and specific numerical values in Figure 5a. The corresponding RMSEP values of these models during the validation phase are similarly annotated in Figure 5b.

Continuing the examination of Figure 5, we identify the optimal models for each type of spectra as those yielding the lowest RMSECV value. For example, the NIRdr model developed under the SNV pretreatment condition delivers the lowest RMSECV value (0.211%) among its counterparts, hence considered the optimal model for NIRdr spectra. A similar approach was used to identify the optimal models for the remaining four types of spectra. The specific numerical values depicted in Figure 5b represent the RMSEP values of the five optimal models during validation. It is noteworthy that the optimal model might not always exhibit the lowest RMSEP value. This could be attributed to potential differences between the calibration and validation datasets.

Table 4 presents detailed information on the five optimal models constructed based on the five different spectra mentioned above. The table includes information on the pretreatment conditions, spectral ranges, latent variables (LVs), and calibration and validation results corresponding to these optimal models. For detailed information on models under all other pretreatment conditions, please refer to Part 5 of the Supplementary Material.

As detailed in Table 4, the five optimal models all performed reliable calibration and verification results, and their R^2^_cv_ and R^2^_v_ were both higher than 0.8, which indicated that these five models could measure the cellulose content of rice bran layer and could be used to screen rice germplasm with cellulose content variation in the bran layer. Please refer to Part 6 of the Supplementary Material for the scatterplots of reference and predicted cellulose content for the calibration and validation sets of these five optimal models.

**Figure 6:**
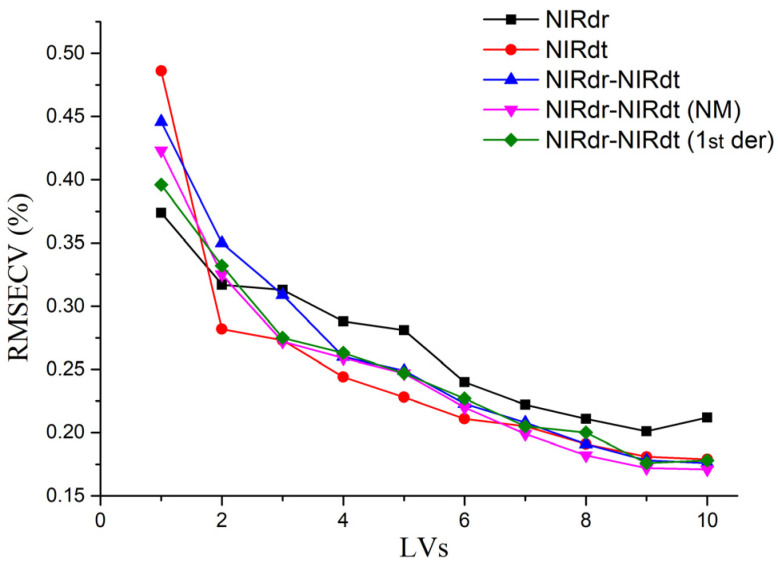
The relationship between RMSECV and LVs in the five optimal models.

The number of Latent Variables (LVs) shown in Table 4 was chosen to balance the need for low RMSECV values with the avoidance of overfitting. Figure 6 illustrates the relationship between the number of Latent Variables (LVs) used in the five optimal models and the corresponding RMSECV values. The principle for selecting the number of LVs is that it should not exceed one-tenth of the number of calibration set samples. Therefore, the maximum number of LVs chosen in this study is nine.

In general, well-performed models need to have low RMSECV values in addition to high R^2^ values. It can be seen from Table 4 that the NIRdr-NIRdt (1st der) model has the lowest RMSECV value (0.176%) under the condition of the MSC pre-treatment, followed by the NIRdr-NIRdt model and the NIRdr-NIRdt (NM) model, and finally the NIRdt model and the NIRdr model. For the validation, the NIRdr-NIRdt (1st der) model showed the lowest RMSEV value (0.169%) under the condition of the MSC pretreatment, followed by the NIRdr-NIRdt model and the NIRdr-NIRdt (NM) model, and finally the NIRdt model and the NIRdr model. The results showed that the NIRdt model showed better prediction performance than the NIRdr model. Furthermore, the model constructed using the fused spectra of NIRdr and NIRdt showed better prediction performance than the NIRdr model and the NIRdt model. Among the three optimal models constructed using fused spectra, the optimal model constructed using the fused spectra NIRdr-NIRdt (1st der) has the best prediction performance.

### 3.5. Spectral Ranges of the Optimal Models

**Figure 7:**
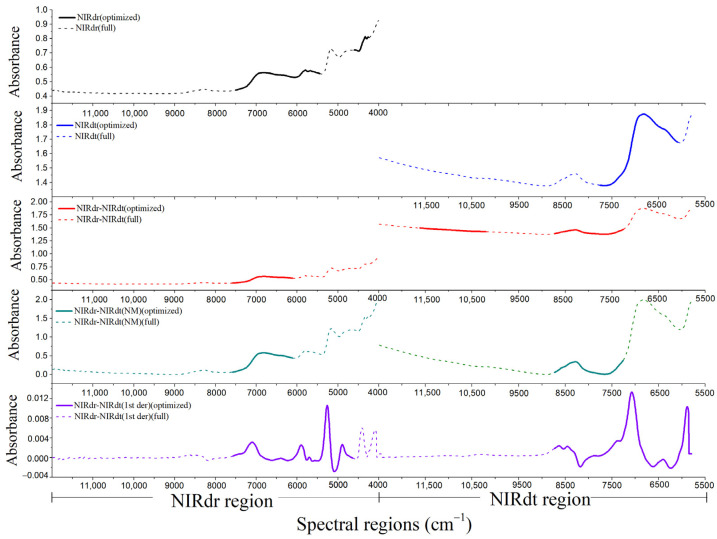
Spectral ranges used by the optimal rice bran layer cellulose content models. In this figure, the black, blue, red, light cyan, and purple dashed lines are the average spectra of NIRdr, NIRdt, NIRdr-NIRdt, NIRdr-NIRdt (NM), and NIRdr-NIRdt (1st der) of the rice bran layer samples, respectively. In contrast, the solid lines of corresponding colors represent the spectral ranges used by the five optimal rice bran layer cellulose content models.

The spectral ranges used by the five optimal rice bran layer cellulose content models, which are constructed based on the NIRdr, NIRdt, NIRdr-NIRdt, NIRdr-NIRdt (NM), and NIRdr-NIRdt (1st der) spectra, respectively, are shown in Figure 7. These optimal models are consistent with those in Table 4, with the lowest RMSECV.

It can be seen from Figure 7 that the primary spectral range engaged by the optimal NIRdr model covers the spectral region of 7500–5400 cm^−1^ (indicated by a solid black line), while the spectral range used by the NIRdt model spans the spectral region of 7800–6100 cm^−1^ (indicated by a solid blue line). There is a significant overlap of the spectral ranges utilized by the three fusion models with those employed by the NIRdr and NIRdt models. The main overlapping ranges comprise the bands within the 7500–6109 cm^−1^ interval in the NIRdr region, as well as the bands falling between 7752–7259 cm^−1^ in the NIRdt region. These overlapping ranges contain some segments associated with cellulose molecule absorption, such as the bands near 1356 nm (7375 cm^−1^, the second overtone of the O–H stretch vibration), 1587 nm (6300 cm^−1^, the first overtone of the C-H stretch vibration) and 1428–1641 nm (7000~6700 cm^−1^, the first overtone of the O–H stretch vibration). The spectral ranges used in this study overlap with those used in Liu’s study, which are 7620–7440 cm^−1^, 7080–6900 cm^−1^, and 5810–5450 cm^−1^ [41].

By comparing the spectral ranges used in the above five optimal models, it can be seen that the spectral ranges used in the optimal fusion models were observed in both the NIRdr and NIRdt regions. These bands contain cellulose-related absorption peaks to varying degrees, and these bands overlap with the bands used in the optimal NIRdr and NIRdt models. The performance of the fusion model is better than that of the NIRdr and NIRdt models, indicating that the NIRdr and NIRdt bands used in the fusion model contain complementary information. Thus, the fusion analysis of the spectra is beneficial to the improvement of the prediction accuracy of the model, which is consistent with Xu’s study [27].

### 3.6. External Prediction of the Models

The above five optimal rice bran layer cellulose content models were used to predict the cellulose content of 20 external prediction samples and then compared with the reference values obtained by the optimized PDO method. The external prediction results of the models are shown in Table 5. Please refer to Part 7 of the Supplementary Material for detailed data and corresponding scatter plots.

According to Table 5, the NIRdr-NIRdt (1st der) model showed the best prediction performance (RMSEP = 0.213%), followed by the NIRdr-NIRdt (NM) model, then the NIRdt and NIRdr-NIRdt models, while the NIRdr model showed the worst prediction performance, with RMSEP higher than 0.3% and R^2^_p_ lower than 0.8%.

**Figure 8:**
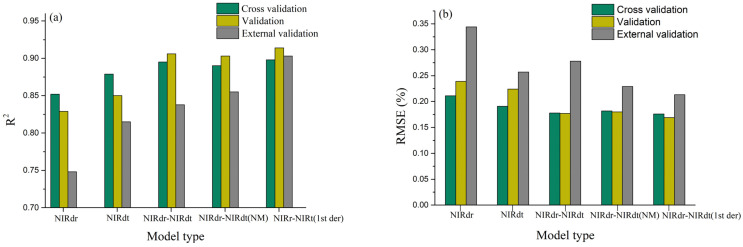
R^2^ (**a**) and RMSE (**b**) of model Cross-validation, Validation, and External Prediction.

The calibration (cross-validation), validation, and external prediction results of the above five optimal models were compared, including the comparison of R^2^ values (Figure 8a) and RMSE (RMSECV or RMSEV or RMSEP) values (Figure 8b). Compared with the calibration and verification results of the above five optimal models, the prediction performance of the five models showed different degrees of deterioration in external prediction, which was manifested by the decrease of R^2^ value and the increase of RMSE value. Among them, the R^2^ value and RMSE value of the NIRdr-NIRdt (1st der) model vary the least, indicating that the model has the strongest stability. However, the R^2^ value and RMSE value of the NIRdr model vary the most, indicating that the stability of the model is the weakest. In addition, when comparing the calibration and validation results of the models, the variation range of the R^2^ value and RMSE value of the NIRdt model is greater than those of the NIRdr-NIRdt model. However, when comparing the validation and external prediction results of the models, the variation range of the R^2^ value and RMSE value of the NIRdt model is smaller than those of the NIRdr-NIRdt model, which indicates that the NIRdt model has a stronger generalization ability than the NIRdr-NIRdt model and can better predict the cellulose content in unknown rice bran layer samples.

Moreover, in this study, we conducted a two-tailed t-test to analyze the relationship between the chemical reference cellulose content of the external prediction set and the cellulose content predicted by various models. In the NIRdt, NIRdr-NIRdt (NM), and NIRdr-NIRdt (1st der) models, there were no statistically significant differences between the predicted results and the chemical reference values, with corresponding *p*-values of 0.75, 0.47, and 0.15, respectively. However, in the NIRdr and NIRdr-NIRdt models, we observed significant differences between the predicted values and the chemical reference values, with respective *p*-values of 0.026 and 0.017.

These findings suggest that although the NIRdr and NIRdr-NIRdt models demonstrate low RMSEP values, their generalizability is limited, potentially posing challenges in practical application. In contrast, the NIRdt, NIRdr-NIRdt (NM), and NIRdr-NIRdt (1st der) models showing no significant discrepancies between their predicted results and the chemical reference values, demonstrate considerable potential for application. They might be particularly advantageous for implementation in practical breeding practices, with the NIRdr-NIRdt (1st der) model exhibiting the most prominent potential among them.

### 3.7. Rice Varieties with Low Cellulose Content in Bran Layer Screened by NIRS

According to Table S2, located in Part 7 of the Supplementary Material, the range of cellulose content in the external prediction set spans from 3.10% to 4.78%. Given that over half of the samples in this prediction set are mutants obtained by heavy ion mutagenesis of the japonica rice variety Wuyunjing 7 (W7), we consequently established the chemical reference cellulose content and the predicted value of W7’s bran layer (4.26%, and 4.14% respectively) as a benchmark, identifying samples with fluctuations in cellulose content exceeding 0.43% as rice varieties with low or high cellulose levels. Table 6 presents a subset of the screening results obtained through chemical reference values and predicted values, with the predicted values acquired through the NIRdr-NIRdt (1st der) model. Table 6 illustrates a selection of results obtained via chemical reference values and predicted values, with the latter derived from the NIRdr-NIRdt (1st der) model. Notably, rice variety O2 demonstrates the lowest cellulose content, with a predicted value of 3.28, representing a decrease of 0.86% compared to wild-type W7 (O2 is a mutant of W7, characterized by its brittle stem).

## 4. Conclusions

We introduced a rapid detection method based on NIRS technology for the cellulose content of the rice bran layer in this study. Our work showcases the potential of this method to screen rice varieties based on variations in bran layer cellulose content. By utilizing an improved rice bran extraction technology, we were able to use de-germinated rice bran as a representative sample of the rice bran layer. This enabled us to determine the cellulose content within the bran layer.

To enhance the accuracy of micro-chemical detection of cellulose, we developed the optimized PDO method, which served as a reliable reference for NIR spectroscopic analysis of cellulose content in the rice bran layer. Furthermore, we employed the fusion technology of NIRdr and NIRdt to optimize the spectral quantitative model of rice bran layer cellulose, achieving rapid and precise detection of its content.

Our results demonstrated that the NIRdr-NIRdt (1st der) quantitative model developed in this study produced satisfactory results, with RMSE (RMSECV, RMSEV, and RMSEP) of cross-validation, validation, and external prediction being 0.176%, 0.169%, and 0.213%, respectively. These findings suggest the potential of NIRS in predicting the cellulose content of the rice bran layer. This is significant as it aids related breeding work and is anticipated to contribute to the palatability improvement and promotion of brown rice.

Despite these promising results, we acknowledge that our study has certain limitations. In particular, our model was constrained by the calibration set for the cellulose range, limited to 2.80% to 4.92%. This restriction presented challenges in accurately predicting the cellulose content for rice bran layer samples with cellulose content outside this range. To address this, future research should focus on expanding the calibration set range, thereby enhancing the model’s applicability.

Moving forward, our method could stimulate further research into the impact of cellulose on the palatability of brown rice and guide the selection of more desirable varieties. These advancements have the potential to improve human health, minimize food waste in the rice processing industry, and, ultimately, strengthen global food security efforts.

## Figures and Tables

**Table 1:** Intra-day precision and inter-day precision test results of the optimized method.

Time	The Parallel Measured Value of the Rice Bran Layer Cellulose Content, %						Mean, %	RSD_r_	RSD_R_
	Value 1	Value 2	Value 3	Value 4	Value 5	Value 6			
Day1	3.86	3.89	3.80	3.91	3.86	3.86	3.86	0.96%	0.70%
Day2	3.84	3.86	3.82	3.83	3.87	3.86	3.84	0.51%	
Day3	3.88	3.84	3.81	3.82	3.84	3.84	3.84	0.63%	

**Table 2:** The mean value, SD, and RSD of the determination results.

Sample	Original PDO Method			Optimized PDO Method		
	Mean (%)	SD (%)	RSD	Mean (%)	SD (%)	RSD
A	3.57	0.097	2.73%	3.38	0.058	1.73%
B	3.62	0.161	4.45%	3.54	0.057	1.61%
C	3.73	0.144	3.86%	3.57	0.075	2.10%
D	3.69	0.061	1.65%	3.74	0.054	1.45%
E	4.58	0.051	1.12%	4.38	0.061	1.39%
F	4.30	0.155	3.60%	4.52	0.011	0.25%
G	4.63	0.092	1.99%	4.75	0.080	1.69%
H	3.00	0.116	3.85%	2.98	0.030	1.00%
I	4.16	0.087	2.10%	4.08	0.065	1.60%
J	3.59	0.046	1.28%	3.60	0.070	1.95%
K	4.33	0.125	2.88%	4.41	0.059	1.33%
L	4.19	0.155	3.71%	4.15	0.047	1.14%
M	4.06	0.114	2.80%	4.16	0.035	0.84%
N	4.08	0.110	2.70%	4.04	0.041	1.01%
O	4.36	0.145	3.33%	4.41	0.059	1.33%

**Table 3:** Descriptive statistics for the cellulose content detection results of the sample set.

Analyte	Calibration Set					Validation Set				
	N	Range	Mean	SE	SD	N	Range	Mean	SE	SD
Cellulose content (%)	99	2.80–4.92	3.89	0.054	0.55	42	2.81–4.89	3.83	0.086	0.57

Note: N: sample number; SE: standard error; SD: standard deviation.

**Table 4:** The detailed information on the five optimal models.

Spectral Type	Pretreatment	Spectral Range (cm^−1^)	Cross-Validation		Validation Set		LVs
			R^2^_CV_	RMSECV/%	R^2^_v_	RMSEV/%	
NIRdr	SNV	7506–5446; 4605–4242	0.852	0.211	0.829	0.239	8
NIRdt	1st der + SNV	7752.9–6094	0.879	0.191	0.850	0.224	8
NIRdr-NIRdt	1st der + SNV	7583.1–6109.7; 8732–7259; 11,671.7–10,198.3	0.895	0.178	0.906	0.177	9
NIRdr-NIRdt (NM)	1st der + SNV	7583.1–6109.7; 8732–7259	0.890	0.182	0.903	0.18	8
NIRdr-NIRdt (1st der)	MSC	7583.1–4636.2; 8732–5793	0.898	0.176	0.914	0.169	9

Note: The spectral range of the NIRdr region is marked in red text, while the spectral range of the NIRdt region is marked in blue text. R^2^_cv_: determination correlation of cross-validation; RMSECV: root mean squares of cross-validation; R^2^_v_: determination correlation of validation; RMSEV: root mean squares of validation; LVs: latent variables; NIRdr: Near-infrared diffuse reflectance; NIRdt: Near–infrared diffuse transmittance.

**Table 5:** External prediction results of NIR models.

Model Type	Sample Number	R^2^_p_	RMSEP/%
NIRdr	20	0.748	0.344
NIRdt	20	0.815	0.257
NIRdr-NIRdt	20	0.838	0.278
NIRdr-NIRdt (NM)	20	0.855	0.229
NIRdr-NIRdt (1st der)	20	0.903	0.213

Note: R^2^_p_: determination correlation of prediction; RMSEP: root mean square errors of prediction.

**Table 6:** Screening results of rice varieties with different levels of bran layer cellulose content.

Cellulose Content Level	Sample Name	Reference Cellulose Content (%)	Predicted Cellulose Content (%)
Low	O1	3.15	3.42
	O2	3.10	3.28
	O5	3.19	3.30
	O9	3.35	3.51
	O17	3.22	3.48
Normal	O7	4.20	4.13
	O13	4.26	4.37
	**W7**	**4.26**	**4.14**
High	O19	4.74	4.58

Note: The bold part was the control group (Wild type variety W7).

## Data Availability

The data are available upon reasonable request from the corresponding author for non-commercial use.

## Supplementary Materials

The following supporting information can be downloaded at: https://www.mdpi.com/article/10.3390/foods12162997/s1, Figure S1: The thickness of 5 mg, 10 mg, 15 mg, and 20 mg rice bran layer samples in the sample cup used for DT measurements; Figure S2: NIRdt spectra collected using the Bruker MPA, corresponding respectively to the rice bran layer powder samples of (a–c) 5 mg, (d–f) 10 mg, (g–i) 15 mg, and (j–l) 20 mg. Within each row of images, from left to right, are the single-channel spectra of the background, the single-channel spectra of the samples, and the absorbance spectra of the samples; Figure S3: Cross-validation, and Validation of NIR models for cellulose: predicted vs. reference value scatter plot. Scatter plots illustrating the correlation between predicted and chemical reference values for the calibration sets of NIRdr, NIRdt, NIRdr-NIRdt, NIRdr-NIRdt (NM), and NIRdr-NIRdt (1st der) models are respectively presented in Figure S1a, S1c, S1e, S1g, and S1i. Conversely, Figure S1b, S1d, S1f, S1h, and S1j respectively depict scatter plots correlating predicted values and chemical reference values for the corresponding validation sets of these models.; Figure S4. Scatter Plots of Predicted vs. Reference Values for External Prediction of NIR Cellulose Models. Each plot represents the correlation between predicted and chemical reference values for the respective models: (a) NIRdr, (b) NIRdt, (c) NIRdr-NIRdt, (d) NIRdr-NIRdt (NM), and (e) NIRdr-NIRdt (1st der).; Table S1: Determination results of seven samples by the optimized PDO method and the original PDO method; Table S2. The calibration and validation results of models.; Table S3. Predicted and Reference cellulose content from the NIR models for the external prediction Set.
