# Supplementary material for: A Rapid and Accurate Quantitative Analysis of Cellulose in the Rice Bran Layer Based on Near-Infrared Spectroscopy
Source: Foods. 2023 Aug 9;12(16):2997. doi: 10.3390/foods12162997 (PMC10453377; doi:10.3390/foods12162997)
Supplement: Supplementary file 1 [file foods-12-02997-s001.zip › foods-2495309-supplementary.pdf]

Supplementary Materials

Part 1. Methodology for Rice Bran Sample Acquisition

The sample of rice bran layer utilized in this study is de-germinated rice bran. As a byproduct of processing brown rice into milled rice, rice bran typically consists of the bran layer, germ, and a small quantity of endosperm. However, under carefully regulated laboratory conditions, it is possible to isolate rice bran primarily comprising the bran layer. This isolated product serves as our rice bran layer sample for cellulose content determination. Further details of this process are elucidated in this part.

Initially, the rice was de-husked by using a JLGJ-4.5 paddy rice seed huller (Taizhou Grain Instrument Factory, China) to yield brown rice.

Subsequently, a certain weight (20-20.5g) of brown rice was put into a rice mill. The milling duration was calibrated to limit the endosperm content in the rice bran as much as possible. It is imperative to note that the milling duration should be tailored based on the specific rice mill utilized. For the JNMJ-3 rice mill (Taizhou Grain Instrument Factory, China) used in this study, the optimal milling duration was determined to be 50 seconds.

Following milling, the rice bran was passed through a 40-mesh sieve to eliminate germs and broken rice kernels within the rice bran. This step facilitated the acquisition of rice bran primarily composed of the bran layer with a trace of endosperm, which is called de-germinated rice bran.

Finally, the de-germinated rice bran was regarded as the rice bran layer sample, suitable for subsequent quantitative cellulose analysis.

Part 2. Experimental procedure of the modified potassium dichromate oxidation (PDO) method

The detailed steps for the experimental procedure of the modified potassium dichromate oxidation (PDO) method are delineated as follows:

(1) Preparation of reagent solutions: A variety of reagent solutions are formulated, including an ammonium ferrous sulfate solution which ought to be prepared no more than one week before its application. It is vital to determine the titration factor (K) of this solution on the day of its use. This ammonium ferrous sulfate solution is then utilized to titrate a 10 mL potassium dichromate solution with an equivalent concentration of 0.01N, the volume of ammonium ferrous sulfate solution used is recorded as "y" mL. The magnitude of the value of K is calculated using formula 1.

$$K= (0.01*10)/y \qquad (1)$$

(2) **Sampling**: A rice bran layer sample of 0.05 to 0.06g is weighed and placed into a test tube.

(3) **Digestion**: A 5mL mixture of nitric acid and acetic acid (volume ratio of 1:1) is introduced into the test tube containing the sample. Following thorough mixing, the test tube is placed in a boiling water bath for 30 minutes with periodic agitation. Upon

completion of the boiling water bath treatment, the test tube is allowed to cool at room temperature.

(4) **Centrifugation**: The contents of the test tube are transferred to a 10 mL centrifuge tube labeled A. The test tube is then rinsed with 1 mL of the nitric-acetic acid mixture, and the rinse is added to the centrifuge tube A. Centrifugation is conducted at 12,000 r/min for 15 minutes, after which the supernatant is discarded.

(5) **Centrifuge Washing**: Distilled water is added up to the 6mL scale line in centrifuge tube A, which is then centrifuged again at 12,000 r/min for 15 minutes. The supernatant is carefully transferred into a new 10 mL centrifuge tube B. Distilled water is again added up to the 6mL scale line in centrifuge tube B, which is then subjected to centrifugation at 12,000 r/min for 15 minutes. The supernatant is discarded, centrifuge tube B is rinsed with a suitable amount of distilled water and its contents are transferred back to centrifuge tube A. This centrifuge washing step is performed a total of three times.

(6) **Oxidation Reaction**: Transfer the contents of centrifuge tube A into a clean test tube. Subsequently, introducing 5 mL of a 0.05 N potassium dichromate-concentrated sulfuric acid mixture (volume ratio of 5:4). Following thorough mixing, the test tube is placed in a boiling water bath for 30 minutes with periodic agitation. Upon completion of the boiling water bath treatment, the test tube is allowed to cool at room temperature.

(7) **Titration**: Transfer the contents from the test tube to a 250 mL conical flask, subsequently ensuring the test tube is thoroughly rinsed with a small quantity of distilled water. The rinse is also added to the conical flask. 3 drops of ferroin indicator are added to the conical flask, and the titration is carried out with a 0.05 N ammonium ferrous sulfate solution. When the solution changes from a yellow-brown to bright blue color, it is advisable to reduce the titration speed until the solution reaches a brick-red color, which signals the titration endpoint. The volume (b mL) of ammonium ferrous sulfate solution used is recorded. Simultaneously, a control group titration is conducted with 5mL of a 0.05 N potassium dichromate-concentrated sulfuric acid mixture that has not reacted with the sample. The volume (a mL) of ammonium ferrous sulfate solution used for the control is also recorded.

(8) **Calculation of Cellulose Content**: The cellulose content, denoted as x(%), is calculated using the following formula: $x = 0.675K*(a-b)/n$, where K refers to the titer of the ammonium ferrous sulfate solution (determined before titration with a range in this study from 0.4988 to 0.5013), a and b are the volumes in mL of the ammonium ferrous sulfate solution used in the control group titration and sample group titration respectively, and n is the weight of the sample in grams.

Part 3. The thickness test for NIRdt.

The thickness of samples of various weights, utilized in the Diffuse Transmittance (DT) mode for this study and contained within the sample cups, is depicted in Figure S1.

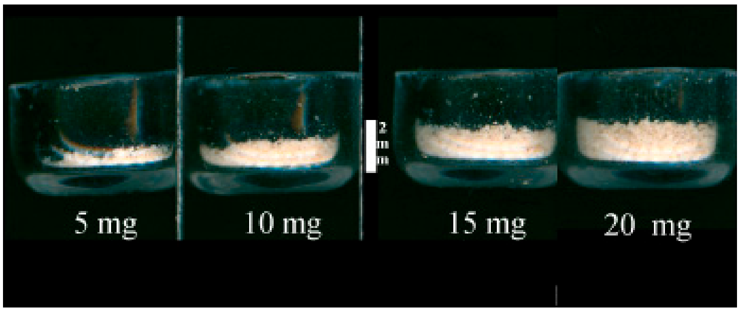

Figure S1. The thickness of 5mg, 10mg, 15mg, and 20mg rice bran layer samples in the sample cup used for DT measurements.

As depicted in Figure S1, the thicknesses of the 5mg, 10mg, 15mg, and 20mg samples within the sample cups are approximately 0.5mm, 1mm, 1.5mm, and 2mm, respectively. Notably, the 5mg sample does not suffice to provide a uniform coverage across the entire base of the sample cup, which could potentially lead to substantial inconsistencies in repeated measurements.

Given these conditions, the corresponding spectral measurements of the samples were made. Figure S2 displays the background single-channel spectra, sample single-channel spectra, and absorbance spectra of rice bran layer samples in diffuse transmittance (DT) mode for 5mg (first row), 10mg (second row), 15mg (third row), and 20mg (fourth row). The absorbance spectra are obtained by calculating the negative logarithm of the ratio between the sample single-channel spectra and the background single-channel spectra.

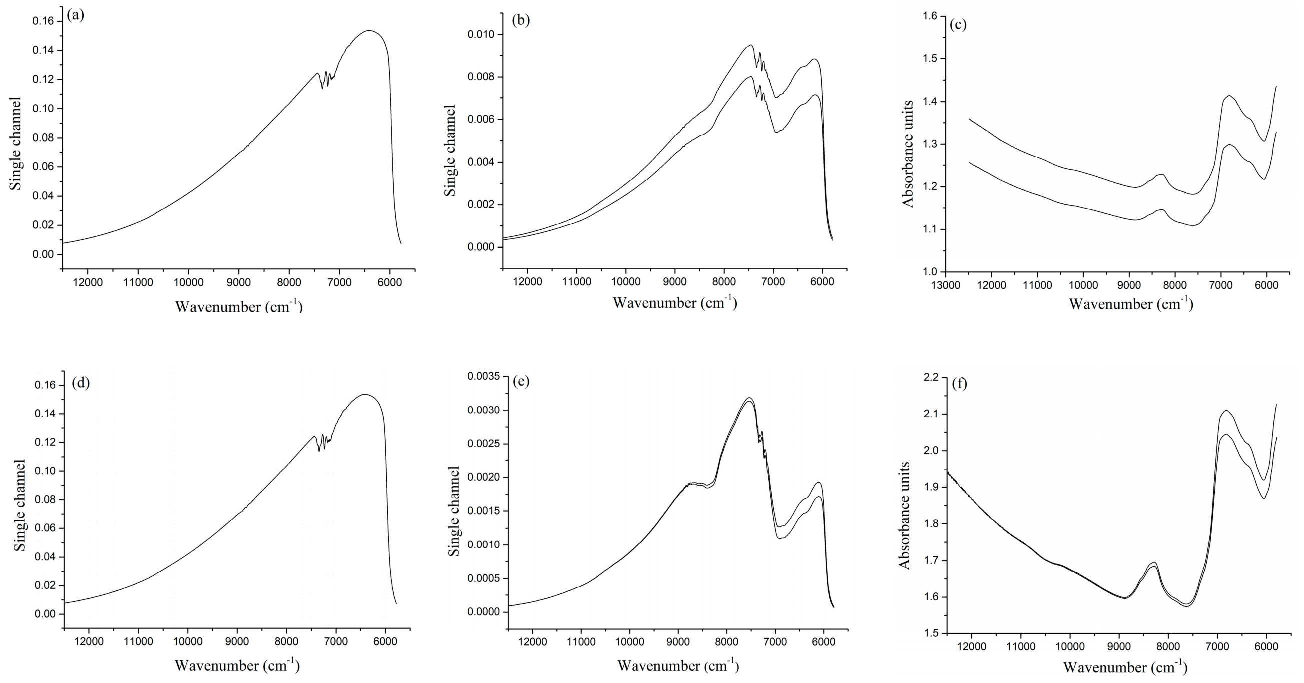

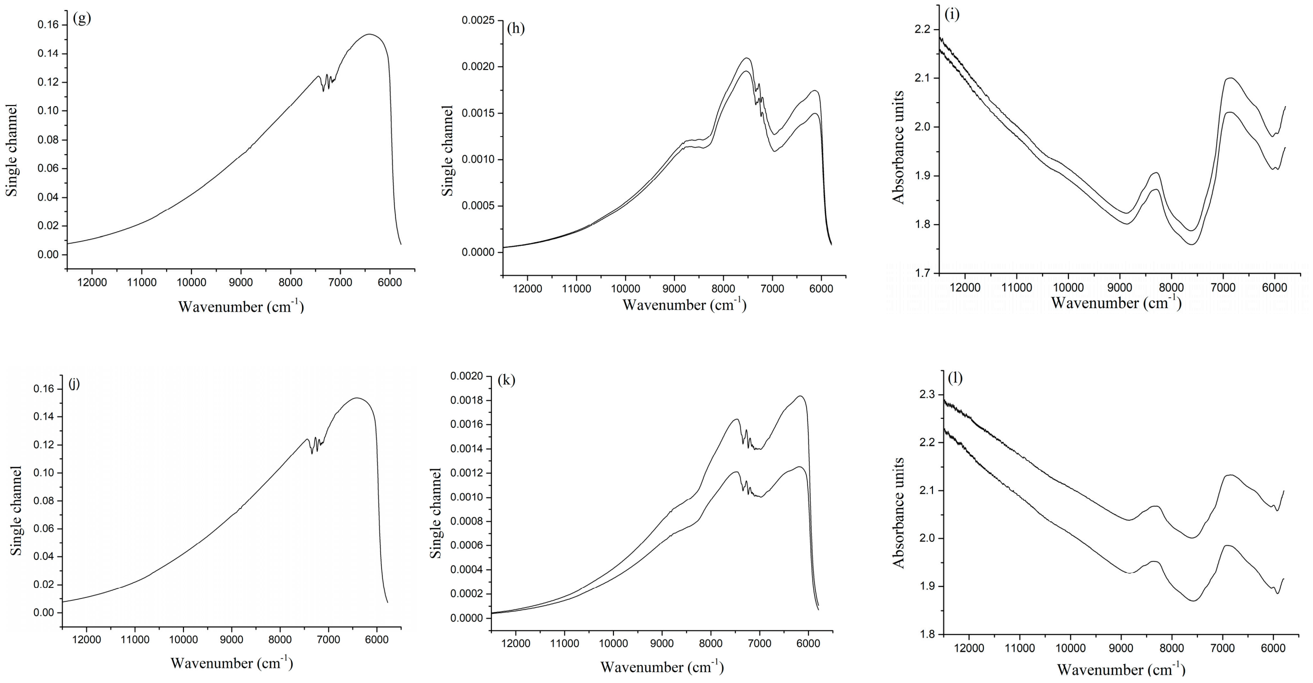

Figure S2. NIRdt spectra collected using the Bruker MPA, corresponding respectively to the rice bran layer powder samples of (a-c) 5mg, (d-f) 10mg, (g-i) 15mg, and (j-l) 20mg. Within each row of images, from left to right, are the single-channel spectra of the background, the single-channel spectra of the samples, and the absorbance spectra of the samples.

The shape of the single-channel spectra of the background reflects the characteristics of the background, which are a combination of air absorption, light emitted by the bulb, and the response characteristics of the detector used under DT mode. The first column of Figure S2 consists of the background's single-channel spectra. A faint absorption peak can be observed around 7300 cm^{-1}, which indicates the presence of minor water vapor absorption within the spectrometer.

Theoretically, given that the collection methods and external conditions remain consistent, the better the reproducibility of the spectra obtained from two spectral acquisitions, the more aligned the trends of these spectra should be. It can be observed that the single-channel spectra of the collected sample tend to undergo significant drift when the sample weight is either too high or too low, leading to terrible reproducibility.

However, when the sample weight is around 10mg, the reproducibility of the sample's absorbance spectra appears to be relatively good. Furthermore, the absorbance values at the absorption peak around 6800cm^{-1} seem to be approaching saturation. Pertaining to this study, when the sample weight is approximately 10mg, the thickness of the sample sufficiently meets the requirements of the study, and the reproducibility of the collected diffuse transmittance spectra is also commendable.

While the sample weight parameter could potentially be further optimized in practical application, the selection of a 10mg sample in this study is reasonable.

Part 4. The detailed determination results of 7 rice bran layer samples.

The detailed determination results obtained by the optimized PDO method and the

original PDO method are shown in Table S1.

Table S1. Determination results of seven samples by the optimized PDO method and the original PDO method.

Sample	Original PDO method				Optimized PDO method			
	Value1 (%)	Value2 (%)	Value3 (%)	RSD (%)	Value1 (%)	Value2 (%)	Value3 (%)	RSD (%)
A	3.68	3.49	3.53	2.73	3.33	3.44	3.37	1.73
B	3.67	3.76	3.44	4.45	3.58	3.47	3.56	1.61
C	3.78	3.56	3.83	3.86	3.52	3.61	3.67	2.10
D	3.64	3.73	3.12	1.65	3.78	3.68	3.75	1.45
E	4.63	4.53	4.58	1.12	4.33	4.43	4.45	1.39
F	4.29	4.15	4.46	3.60	4.51	4.53	4.53	0.25
G	4.68	4.69	4.52	1.99	4.67	4.82	4.80	1.69
H	2.90	2.98	3.13	3.85	2.95	2.97	3.01	1.00
I	4.09	4.26	4.14	2.10	4.01	4.08	4.14	1.60
J	3.55	3.58	3.64	1.28	3.53	3.61	3.67	1.95
K	4.23	4.47	4.29	2.88	4.34	4.43	4.45	1.33
L	4.06	4.36	4.14	3.71	4.17	4.10	4.19	1.14
M	3.93	4.15	4.09	2.80	4.20	4.13	4.16	0.84
N	4.19	4.07	3.97	2.70	4.05	4.00	4.08	1.01
O	4.22	4.51	4.35	3.33	4.45	4.43	4.34	1.33

Note: Value1, Value2, and Value3 are the results of three parallel measurements. RSD is the relative standard deviation. The data marked as red in the table are obvious abnormal data in 3 parallel measurements, which are not included in the calculation of RSD.

Part5. The detailed information of models under various pretreatment.

Table S2. The calibration and validation results of models.

**Spectral type**	**Pretreatment**	**Spectral range (cm^{-1})**	**Cross-validation**		**Validation Set**		**LVs**
			**R^2_{CV}**	**RMSECV/%**	**R^2_V**	**RMSEV/%**	
**NIRdr**	None	7506-5446; 4605-4242	0.838	0.222	0.832	0.237	9
	**SNV**	**7506-5446; 4605-4242**	**0.852**	**0.211**	**0.829**	**0.239**	**8**
	MSC	7506-5446; 4605-4242	0.849	0.214	0.844	0.228	7
	1st der	7506-5446; 4605-4242	0.825	0.23	0.858	0.217	8
	1st der + SNV	7506-5446; 4605-4242	0.834	0.224	0.821	0.244	7
	1st der + MSC	7506-5446; 4605-4242	0.844	0.217	0.872	0.207	6
**NIRdt**	None	7752.9-6094	0.776	0.26	0.829	0.239	8
	SNV	7752.9-6094	0.855	0.21	0.840	0.231	8
	MSC	7752.9-6094	0.856	0.209	0.841	0.23	8
	1st der	7752.9-6094	0.866	0.201	0.840	0.231	8
	**1st der + SNV**	**7752.9-6094**	**0.879**	**0.191**	**0.850**	**0.224**	**8**
	1st der + MSC	7752.9-6094	0.874	0.195	0.849	0.225	8
**NIRdr-NIRdt**	None	9048.8-7575.4; 8732-7259	0.812	0.239	0.794	0.262	9
	SNV	9048.8-7575.4; 8732-7259	0.860	0.206	0.847	0.226	9
	MSC	7583.1-6109.7; 10206-7259	0.877	0.193	0.901	0.182	8

	1st der	7583.1-6109.7; 8732~7259	0.870	0.198	0.898	0.184	9
	**1st der + SNV**	**7583.1-6109.7; 8732-7259; 11671.7-10198.3**	**0.895**	**0.178**	**0.906**	**0.177**	**9**
	1st der + MSC	7583.1-6109.7; 8732-5793; 11671.7-10198.3	0.884	0.187	0.893	0.189	9
**NIRdr-NIRdt**(NM)	None	4643.9-4000; 8732-5793; 12489.4-10198.3	0.842	0.219	0.849	0.225	9
	SNV	9048.8-7575.4; 7266-5793; 12489.4-10198.3	0.843	0.218	0.842	0.23	9
	MSC	9048.8-7575.4; 10206-7259	0.849	0.214	0.860	0.216	8
	1st der	9048.8-7575.4; 10206-7259	0.869	0.199	0.853	0.221	8
	**1st der + SNV**	**7583.1-6109.7; 8732-7259**	**0.890**	**0.182**	**0.903**	**0.18**	**8**
	1st der + MSC	9048.8-6109.7; 10206-7259	0.870	0.198	0.871	0.208	8
**NIRdr-NIRdt**(1st der)	None	**9048.8-7575.4; 11671.7-5793.4**	0.855	0.21	0.873	0.206	9
	SNV	**7583.1-6109.7; 8732-7259; 11671.7-10198.3**	0.895	0.178	0.905	0.178	9
	**MSC**	**7583.1-4636.2; 8732-5793**	**0.898**	**0.176**	**0.914**	**0.169**	**9**
	1st der	**7583.1-6109.7; 10206-7259**	0.864	0.203	0.861	0.216	9
	1st der + SNV	**4643.9-4000; 9048.8-7575.4; 8732-5793; 12489.4-11671.7**	0.857	0.208	0.880	0.2	9
	1st der + MSC	**4643.9-4000;7583.1-6109.7; 8732-5793; 12489.4-11671.7**	0.862	0.204	0.876	0.203	8

Note：The spectral range of the NIRdr region is marked in red text, while the spectral range of the NIRdt region is marked in blue text. R^2_{cv}: determination correlation of cross-validation; RMSECV: root mean squares of cross-validation; R^2_v: determination correlation of validation; RMSEV: root mean squares of validation; LVs: latent variables; NIRdr: Near–infrared diffuse reflectance; NIRdt: Near–infrared diffuse transmittance. The optimal models under each spectral type in the table are boldfaced.

Part 6. Cross-Validation and Validation Scatter Plots

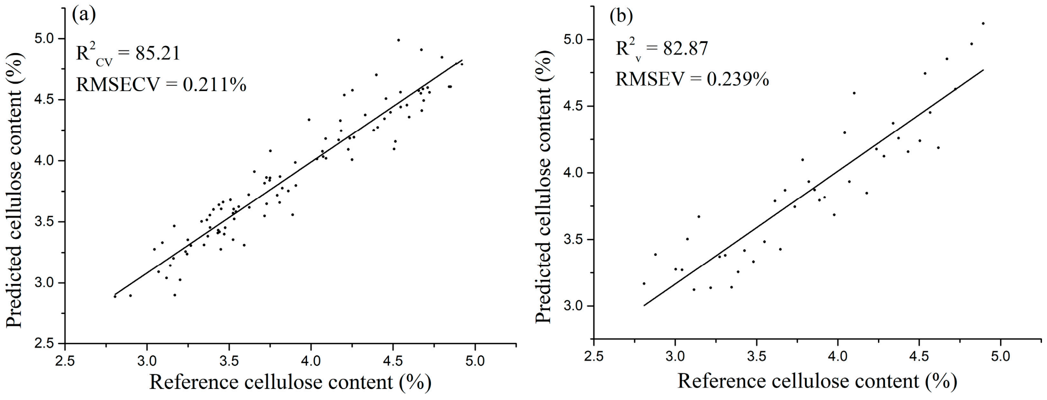

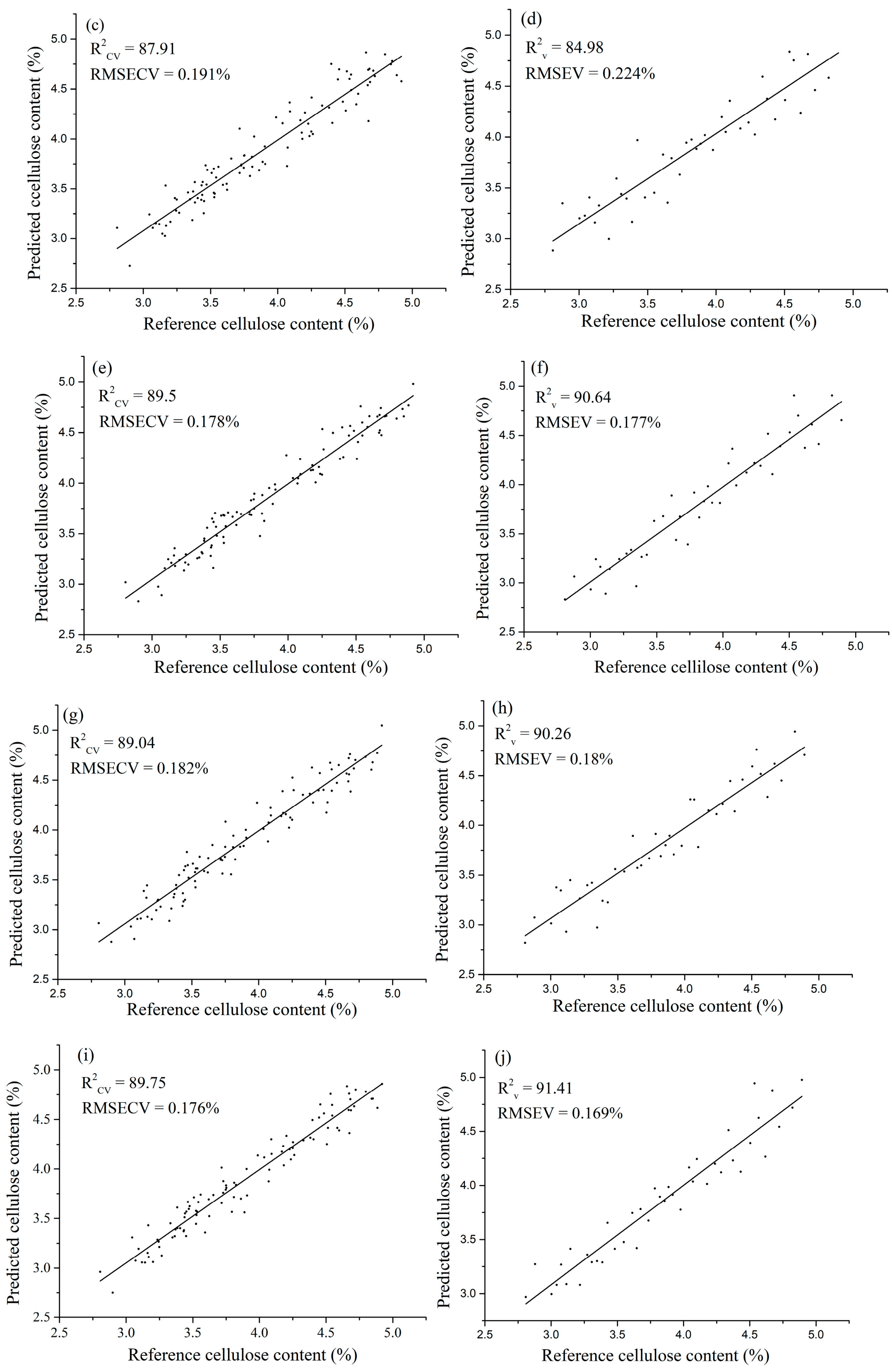

Figure S3. Cross-validation, and Validation of NIR models for cellulose: predicted vs. reference value scatter plot. Scatter plots illustrating the correlation between predicted and chemical reference values for the calibration sets of NIRdr, NIRdt, NIRdr-NIRdt, NIRdr-NIRdt (NM), and NIRdr-NIRdt (1st der) models are respectively presented in figures S1(a), S1(c), S1(e), S1(g), and S1(i). Conversely, figures S1(b),

S1(d), S1(f), S1(h), and S1(j) respectively depict scatter plots correlating predicted values and chemical reference values for the corresponding validation sets of these models.

Part 7. External prediction scatter plots and corresponding data

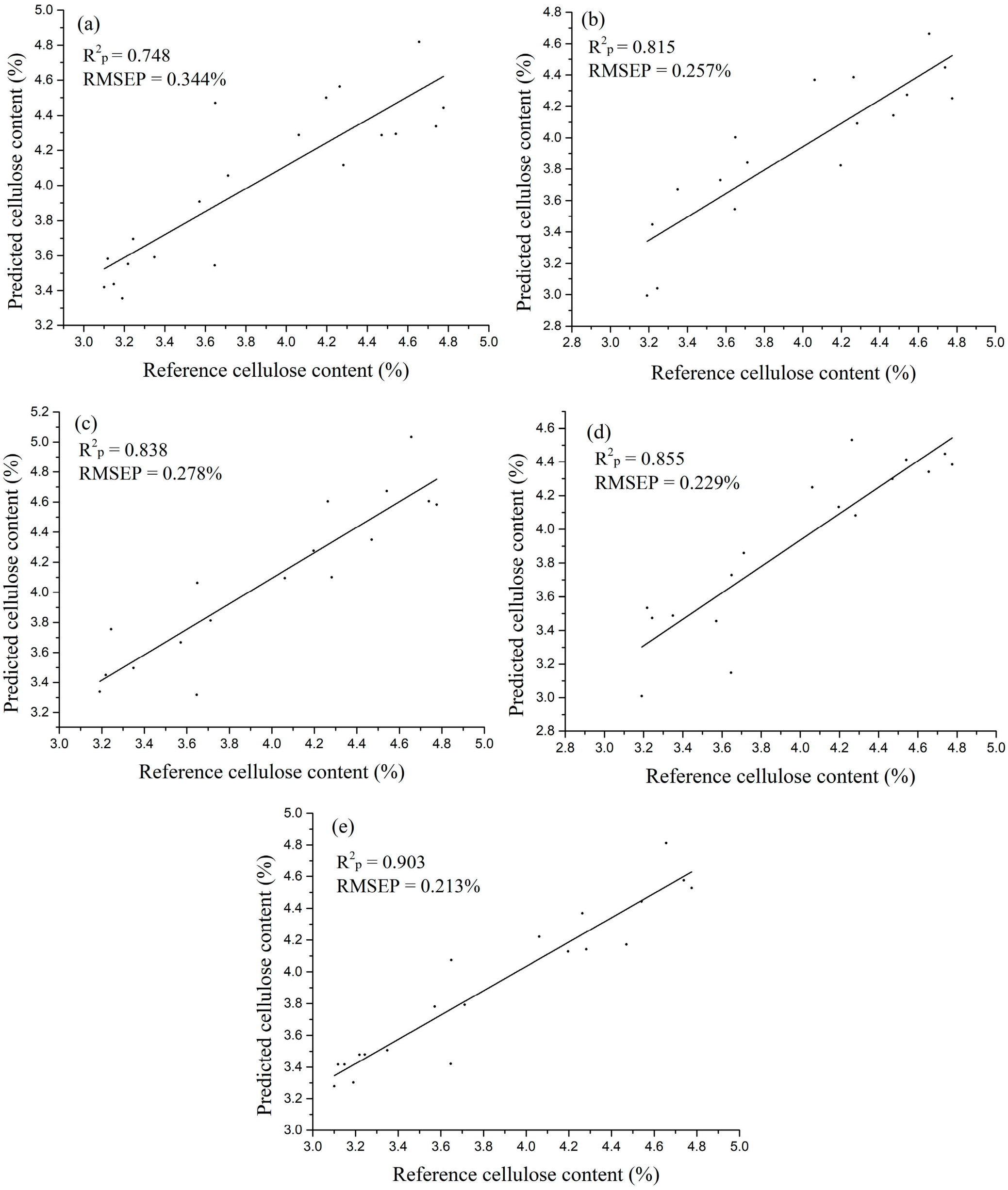

Figure S4. Scatter Plots of Predicted vs. Reference Values for External Prediction of NIR Cellulose Models. Each plot represents the correlation between predicted and chemical reference values for the respective models: (a) NIRdr, (b) NIRdt, (c) NIRdr-NIRdt, (d) NIRdr-NIRdt (NM), and (e) NIRdr-NIRdt (1_{st} der).

Table S3. Predicted and Reference cellulose content from the NIR models for the external prediction Set

**Sample name**	**Reference cellulose content (%)**	**Predicted cellulose content (%)**				
		**NIRdr**	**NIRdt**	**NIRdr-NIRdt**	**NIRdr-NIRdt(NM)**	**NIRdr-NIRdt(1_{st} der)**

O1	3.15	3.44	3.32	3.53	3.28	3.42
O2	3.10	3.42	3.18	3.61	3.11	3.28
O3	3.12	3.58	3.35	3.44	3.21	3.42
O4	3.24	3.70	3.04	3.75	3.47	3.48
O5	3.19	3.36	2.99	3.34	3.01	3.30
O6	4.66	4.82	4.66	5.03	4.34	4.81
O7	4.20	4.50	3.82	4.28	4.13	4.13
O8	3.65	4.47	4.00	4.06	3.73	4.07
O9	3.35	3.59	3.67	3.50	3.49	3.51
O10	4.06	4.29	4.37	4.09	4.25	4.22
O11	3.65	3.54	3.54	3.32	3.15	3.42
O12	3.71	4.06	3.84	3.81	3.86	3.79
O13	4.26	4.56	4.39	4.60	4.53	4.37
O14	4.47	4.29	4.14	4.35	4.30	4.17
O15	4.28	4.12	4.09	4.10	4.08	4.14
O16	3.57	3.91	3.73	3.67	3.46	3.78
O17	3.22	3.55	3.45	3.45	3.53	3.48
O18	4.78	4.44	4.25	4.58	4.38	4.53
O19	4.74	4.34	4.45	4.61	4.45	4.58
O20	4.54	4.29	4.27	4.67	4.41	4.44
